# Comparison and Avoidance of Toxicity of Penetrating Cryoprotectants

**DOI:** 10.1371/journal.pone.0027604

**Published:** 2011-11-16

**Authors:** Edyta A. Szurek, Ali Eroglu

**Affiliations:** 1 Institute of Molecular Medicine and Genetics, Georgia Health Sciences University, Augusta, Georgia, United States of America; 2 Department of Medicine, Medical College of Georgia, Georgia Health Sciences University, Augusta, Georgia, United States of America; 3 Department of Obstetrics and Gynecology, Medical College of Georgia, Georgia Health Sciences University, Augusta, Georgia, United States of America; 4 Cancer Center, Georgia Health Sciences University, Augusta, Georgia, United States of America; Baylor College of Medicine, United States of America

## Abstract

The objective of this study was to elucidate the toxicity of widely used penetrating cryoprotective agents (CPAs) to mammalian oocytes. To this end, mouse metaphase II (M II) oocytes were exposed to 1.5 M solutions of dimethylsulfoxide (DMSO), ethylene glycol (EG), or propanediol (PROH) prepared in phosphate buffered saline (PBS) containing 10% fetal bovine serum. To address the time- and temperature-dependence of the CPA toxicity, M II oocytes were exposed to the aforementioned CPAs at room temperature (RT, ∼23°C) and 37°C for 15 or 30 minutes. Subsequently, the toxicity of each CPA was evaluated by examining post-exposure survival, fertilization, embryonic development, chromosomal abnormalities, and parthenogenetic activation of treated oocytes. Untreated oocytes served as controls. Exposure of MII oocytes to 1.5 M DMSO or 1.5 M EG at RT for 15 min did not adversely affect any of the evaluated criteria. In contrast, 1.5 M PROH induced a significant increase in oocyte degeneration (54.2%) and parthenogenetic activation (16%) under same conditions. When the CPA exposure was performed at 37°C, the toxic effect of PROH further increased, resulting in lower survival (15%) and no fertilization while the toxicity of DMSO and EG was still insignificant. Nevertheless, it was possible to completely avoid the toxicity of PROH by decreasing its concentration to 0.75 M and combining it with 0.75 M DMSO to bring the total CPA concentration to a cryoprotective level. Moreover, combining lower concentrations (i.e., 0.75 M) of PROH and DMSO significantly improved the cryosurvival of MII oocytes compared to the equivalent concentration of DMSO alone. Taken together, our results suggest that from the perspective of CPA toxicity, DMSO and EG are safer to use in slow cooling protocols while a lower concentration of PROH can be combined with another CPA to avoid its toxicity and to improve the cryosurvival as well.

## Introduction

Successful cryopreservation of human oocytes would facilitate treatment of female infertility resulting from cancer therapy and premature ovarian failure, and would avoid many legal and ethical complications of embryo freezing. Furthermore, successful oocyte banking may help with delayed child-bearing, conservation of genetic material of endangered species, and improving livestock breeding. Although the first successful cryopreservation of mouse and human oocytes was achieved, respectively, in the 1970s [1], [2] and 1980s [3], reproducing the initial success of human oocyte cryopreservation has proved to be challenging due to diverse cryoinjuries/stresses caused by exposure to cryoprotective agents (CPAs), cooling, and combination thereof. Such injuries include intracellular ice formation [4], solution effects injury [5], chilling injury [6], depolymerization/disruption of the oocyte cytoskeleton and spindle microtubules [7], [8], premature exocytosis of cortical granules and zona hardening [9], parthenogenetic activation [10], [11], and aneuploidy/polyploidy [8], [12]. The use of intracytoplasmic sperm injection (ICSI) in conjunction with oocyte cryopreservation has overcome the fertilization failure caused by zona hardening and has led to a live birth [13]. Subsequently, encouraging results have been reported using both slow cooling [14], [15] and vitrification techniques [16], [17], [18]. However, further research is needed to address other cryoinjuries and to develop safe, reliable, and efficient cryopreservation protocols.

To survive the cryopreservation process, cells typically require the presence of CPAs, a class of compounds that specifically act to maintain the viability of cryopreserved cells [19]. The protective nature of penetrating CPAs was first discovered through the use of glycerol in 1949 [20]. Later, dimethylsulfoxide (DMSO) [21] and other polyols such as 1,2-propanediol (PROH) and 1,2-ethandiol (also called ethylene glycol, EG) [22], [23] were introduced and successfully used in cryopreservation of many cell types. Concurrently, CPA toxicity has been recognized as a critical barrier to further advancement of the field. In fact, the toxicity of CPAs has been considered as the single most limiting factor to development of successful cryopreservation protocols for challenging cells and tissues [24]. This is particularly true for vitrification protocols that require initial high CPA concentrations to bring samples into a glassy state without ice formation. Consequently, CPA toxicity has been studied mostly from the perspective of vitrification [25], [26], [27], [28], [29], [30], [31], [32], although moderate concentrations of CPAs used in slow cooling protocols could also be toxic [10], [33], [34], [35], [36], [37]. One obvious approach to cope with CPA toxicity would be the use of less toxic CPAs in cryopreservation techniques. Thus far, toxicity studies have yielded inconsistent results with no consensus on the least and most toxic CPA(s). For instance, PROH [26], [32], [38], [39], [40], EG [28], [30], [33], and DMSO [29], [31] have each been found to be the least toxic CPA among those tested while each of these CPAs was also found to be the most toxic in different studies [28], [29], [30], [31], [32], [33], [40]. These discrepancies might be due to differences in experimental protocols, cell type, and species. With the exception of a few [28], [32], [33], most of the toxicity studies combined the CPA exposure with different cryopreservation procedures, further complicating the toxicity outcome. None of the studies compared the toxicity of three widely used penetrating CPAs (i.e., DMSO, PROH, and EG) side-by-side from the perspective of slow cooling protocols. Taken together, the area of CPA toxicity remains one of considerable uncertainty and is ripe for re-evaluation, as stated in a recent review [41].

The objective of this study was to systematically compare the toxicity of the aforementioned three penetrating CPAs (i.e., DMSO, PROH, and EG) from the perspective of slow cooling protocols and to develop a strategy to avoid potential CPA toxicity. Glycerol was excluded due to its inability to adequately permeate oocytes. To decouple the effect of a given cryopreservation protocol, mouse oocytes were exposed to each CPA at different temperatures for different durations of time, without subjecting them to a freeze-thaw cycle. Thereafter, treated oocytes were analyzed for survival, fertilization, embryonic development, as well as for chromosomal abnormalities (ploidy) and parthenogenetic activation compared to untreated controls. Additional experiments were undertaken to circumvent CPA toxicity by combining lower concentrations of CPAs while keeping the total CPA concentration unchanged.

## Materials and Methods

### Reagents and Media

All chemicals were purchased from Sigma (St. Louis, MO) unless otherwise stated. HEPES-buffered Dulbecco's Modified Eagle Medium (DMEM)/F-12 mixture (Gibco, Grand Island, NY) containing 10% fetal bovine serum (FBS) (Gemini Bio-Products, West Sacramento, CA) was used for all oocyte manipulations under air. Dulbecco's Phosphate Buffered Saline (DPBS) (Gibco, Grand Island, NY) containing 10% of FBS served as a base to prepare CPA solutions. Oocytes and embryos were cultured in bicarbonate-buffered Hypermedium [42] containing 4 mg/ml bovine serum albumin (BSA) (Serologicals Proteins Inc., Kankakee, IL) at 37°C under a humidified atmosphere of 5% CO_2_ in air. Dispersion of sperm was performed in a 0.4-ml drop of DMEM. Prior to use, drops of the Hypermedium and DMEM were overlaid by embryo-tested mineral oil (Humco, Texarkana, TX) and equilibrated overnight under a humidified atmosphere of 5% CO_2_ in air.

### Oocyte Isolation

Metaphase II (M II) oocytes were obtained from 5–8 week-old B6D2F1 hybrid mice (C57BL/6NCrl X DBA/2NCrl; NCI, Frederick, MD). This was approved by the Institutional Animal Care and Use Committee at Georgia Health Science University (AUP# BR09-11-267). Superovulation was induced by a combination of 5 IU pregnant mare serum gonadotropin and 2.5 IU human chorionic gonadotropin (hCG) (PG 600, Intervet, Millsboro, DE) followed by 7.5 IU hCG alone 49 hours later. Both hormone solutions were given intraperitoneally. To collect M II oocytes, the oviducts were excised from euthanized mice 14 hours after hCG injection and oocyte-cumulus masses were released from the ampulla. To remove cumulus cells, the oocyte-cumulus masses were exposed to 120 IU/ml of bovine testis hyaluronidase (Type IV-S) at ambient temperature for 3–4 min. Next, the oocytes were washed in DMEM/F-12 with 10% FBS twice and then transferred to Hypermedium for recovery before experimentation. For each experiment, oocytes that were isolated from 3 to 4 females were pooled and then randomly distributed to experimental groups.

### Cryoprotectant Exposure Experiments

To elucidate the toxicity of penetrating CPAs, we conducted a series of comprehensive experiments. The first set of CPA exposure experiments was designed to compare the toxicity of three conventional penetrating CPAs (i.e., DMSO, EG, and PROH) at room temperature (RT, 23°C). To this end, we exposed M II mouse oocytes to a 1.5 M solution of each CPA in PBS+10%FBS for 15 minutes, a time period sufficient to load approximately 1.5 M concentration of each CPA at 23°C. The CPA concentration and exposure time were selected to make them comparable to values typically used for slow cooling protocols of mammalian oocytes. At the end of the exposure period, we stepwise removed the tested CPA by successively transferring treated oocytes to decreasing CPA concentrations (i.e., 1.0 M, 0.5 M, and 0.0 M) in PBS+10%FBS with 5-minute intervals at ambient temperature. After 5-minute holding at the final dilution step, the oocytes were rinsed in a fresh drop of PBS and then washed one more time in Hypermedium before being transferred to fresh culture drops of Hypermedium for a recovery period of 1 hour at 37°C. Subsequently, the toxicity of all three CPAs to M II oocytes was assayed by examining their effect on post-exposure survival, fertilization, blastocyst formation, chromosomal abnormalities, and parthenogenetic activation. To do so, some of the oocytes were either inseminated or artificially activated as described below while others were cultured overnight without any further manipulation to determine parthenogenetic activation rates after exposure to each CPA. Oocytes that were not exposed to sperm but cleaved after overnight culture were considered as parthenogenetically activated. Untreated M II oocytes that were maintained in Hypermedium at 37°C served as controls. Another control group consisted of oocytes that were kept in PBS+10% FBS at RT (23°C) for ∼30 minutes, and then transferred to the culture medium along with CPA-treated oocytes.

The second set of experiments was carried out to test time- and temperature-dependence of the CPA toxicity by exposing MII oocytes to 1.5 M solution of each CPA at 37°C for 0, 15, and 30 minutes. The rationale for performing this set of exposure experiments at 37°C was two-fold: (i) to probe the temperature-dependence of the CPA toxicity (23°C vs. 37°C); and (ii) to further challenge the oocytes based on results of the first set of experiments at 23°C. After the CPA exposure and recovery at 37°C for 1 hour, the toxicity of the three CPAs was assessed by evaluating post-exposure survival, fertilization, blastocyst formation, chromosomal abnormalities, and parthenogenetic activation with respect to non-treated controls.

The third set of experiments was aimed to address the dose-dependence of CPA toxicity. We have not tested higher concentrations (>1.5 M) of the CPAs based on published results showing that 2.0 M concentrations of all three CPA have a significant toxic effect on mouse M II oocytes even after a short exposure period of 3 minutes at RT [28]. Also, it was not necessary to test lower concentrations of DMSO and EG based on the results of the first two sets of experiments. Consequently, we focused our experiments on PROH and tested its toxicity by exposing mouse oocytes to half (0.75 M) of its typical concentration at both RT and 37°C. Untreated oocytes kept in Hypermedium at 37°C served as controls.

In the fourth and final set of experiments, we attempted to alleviate the CPA toxicity by combining lower concentrations (i.e., 0.75 M) of PROH and DMSO based on previous results while keeping the combined total concentration of both CPAs at 1.5 M. Mouse oocytes were exposed to the combined solution of PROH and DMSO at both RT and 37°C for 15 minutes, and their post-exposure survival, fertilization, blastocyst formation, chromosomal abnormalities, and parthenogenetic activation were examined with respect to non-treated controls. In addition, we carried out cryopreservation experiments to test whether combining lower concentrations (i.e., 0.75 M) of PROH and DMSO provides adequate cryoprotection. To do so, M II oocytes were cryopreserved in the presence of 0.75 M PROH+0.75 M DMSO in PBS containing 10% FBS and their post-thaw survival was compared to those cryopreserved in the presence of 1.5 M DMSO alone, as described next.

### Oocyte Cryopreservation

M II oocytes were randomly allocated to two groups and loaded with either 0.75 M PROH+0.75 M DMSO or 1.5 M DMSO at RT as described above. During the last few minutes of CPA loading, oocytes were aspirated into 0.25-cc plastic straws (TS Scientific, Perkasie, PA) and placed in a programmable freezer (KRYO 10 Series III, Planer, Middlesex, UK) at 0°C and cooled to −6°C at a rate of 2°C/min. After seeding of extracellular ice and holding at −6°C for 10 min, the straws were first cooled to −60°C at 0.5°C/min and then −80°C at 5°C/min where they were held for 2 min before plunging into liquid nitrogen. Thawing was done by introducing the straws into the controlled-rate freezer at −80°C and warming them up to 0°C at 8°C/min. Subsequently, the contents of the straws were released into an empty dish, and then CPAs were diluted by transferring oocytes to successive lower CPA concentrations (i.e., either 0.50 M PROH+0.50 M DMSO, 0.25 M PROH+0.25 M DMSO, and PBS+10% FBS or 1.0 M DMSO, 0.5 M DMSO, and PBS+10% FBS) at RT with 10-min intervals. Next, oocytes were rinsed in PBS+10% FBS one more time before transferring to Hypermedium for incubation at 37°C. Post-thaw survival of cryopreserved oocytes was assessed after >1 h of culture at 37°C by morphological criteria that included translucent appearance of cytoplasm, integrity of the plasma membrane and the zona pellucida, and the size of the perivitelline space.

### In Vitro Fertilization and Embryo Culture

In vitro fertilization (IVF) and culture of inseminated oocytes were carried out in Hypermedium at 37°C under a humidified atmosphere of 5% CO_2_ in air as described elsewhere [43]. Briefly, sperm were obtained from the cauda epididymides of a mature (4–6 months old) BDF1 male mice (NCI). The cauda epididymides were dissected and placed in a large drop (0.4 ml) of pre-equilibrated DMEM. Sperm were released into the medium by gently puncturing the epididymides with a hypodermic needle and were allowed to disperse at 37°C for 15 min. After dispersion, an appropriate volume of the sperm suspension was added to each insemination drop containing Hypermedium with BSA supplementation to give a final concentration of 1–2×10^6^ sperm/ml. The insemination drops were then incubated for 1–2 h to capacitate sperm before introducing untreated and treated oocytes. After 5–6 h of incubation with sperm, all oocytes were washed twice in Hypermedium and then cultured in fresh drops of the same medium. Cleavage to the two-cell stage was examined after overnight culture while development to the blastocyst stage was evaluated after an additional 4 days of culture. Fertilization and blastocyst formation rates were calculated based on the number of surviving oocytes and two-cell embryos, respectively.

### Artificial Activation of M II Oocytes and Chromosomal Analysis

Since depolymerization/disruption of meiotic spindle microtubules and microfilaments by penetrating CPAs may result in dispersion and improper segregation of chromosomes after activation of oocytes by sperm, it is important to address the consequences of the cytoskeletal toxicity of penetrating CPAs. To this end, we decided to analyze chromosomal abnormalities after artificial activation because this approach eliminates any contribution of sperm-originated chromosomal abnormalities. After a post-exposure recovery period of 1 h, both CPA- exposed and untreated control oocytes were artificially activated by incubating them in a modified calcium- and magnesium-free Hypermedium containing 10 mM SrCl_2_ at 37°C for 5–6 h. At the end of the incubation period, all oocytes were washed three times in regular Hypermedium and microscopically examined for evidence of activation. Oocytes that displayed the 2^nd^ polar body and a pronucleus were considered as activated and further cultured for chromosomal analysis as described below.

Activated oocytes were cultured overnight in Hypermedium supplemented with 1 µg/mL colcemid (Gibco, Grand Island, NY) to arrest cell division at metaphase. Thereafter, chromosome spreads were prepared using a modified gradual fixation/air drying method [44] as follows: First, oocytes were exposed to acidic Tyrode's solution to remove zona pellucida, and then transferred to a hypotonic citrate solution (0.9% sodium citrate:30% FBS; 3∶1) for 30 min. Next, a single oocyte was transferred to a small drop of the hypotonic solution on a grease-free slide; fixative I, consisting of methanol (Fisher chemicals), glacial acetic acid (Across organic, New Jersey), and distilled water (5∶1∶4), was gradually added to the drop. After 5 minutes, the fixed oocyte was transferred to a new grease-free slide in a minimum volume of fixative I. Following mounting, the oocyte was covered with a gentle flow of fixative II (methanol∶glacial acetic acid, 3∶1), and the slide was immediately placed in a Coplin jar containing fixative II for 10 min. Afterwards, the slide was dipped into fixative III (methanol∶ glacial acetic acid∶distilled water, 3∶3∶1) for 1 min and then dried by blowing warm air. Finally, chromosome spreads were stained with 5% Giemsa (EMD Chemicals, La Jolla, CA) for 15 min to assess chromosomal normality.

### Statistical Analysis

Experiments in each series were repeated at least three times using at least 10 oocytes per experimental group in each replicate. The total number of oocytes (n) used in each experimental group was shown in figures. The data on post-exposure survival, fertilization, and blastocyst rates were analyzed by ANOVA with Tukey's Multiple Comparison Test using GraphPad Prism (GraphPad Software, Inc., San Diego, CA). Before ANOVA, arcsine transformation was performed on proportional data. The data on parthenogenetic activation, ploidy, and post-thaw survival were analyzed by Fisher's Exact Test using GraphPad Prism. Differences between the groups were considered statistically significant when the *p*-value was less than 0.05. Data reported are means of survival, fertilization, development, and parthenogenetic activation rates with error bars representing standard error of mean (SEM) while the ploidy rates represent percentage of total number of euploid eggs pooled from three replicates. Therefore, the ploidy rates do not have error bars.

## Results

### Toxicity of CPAs at Room Temperature

**Figure 1:**
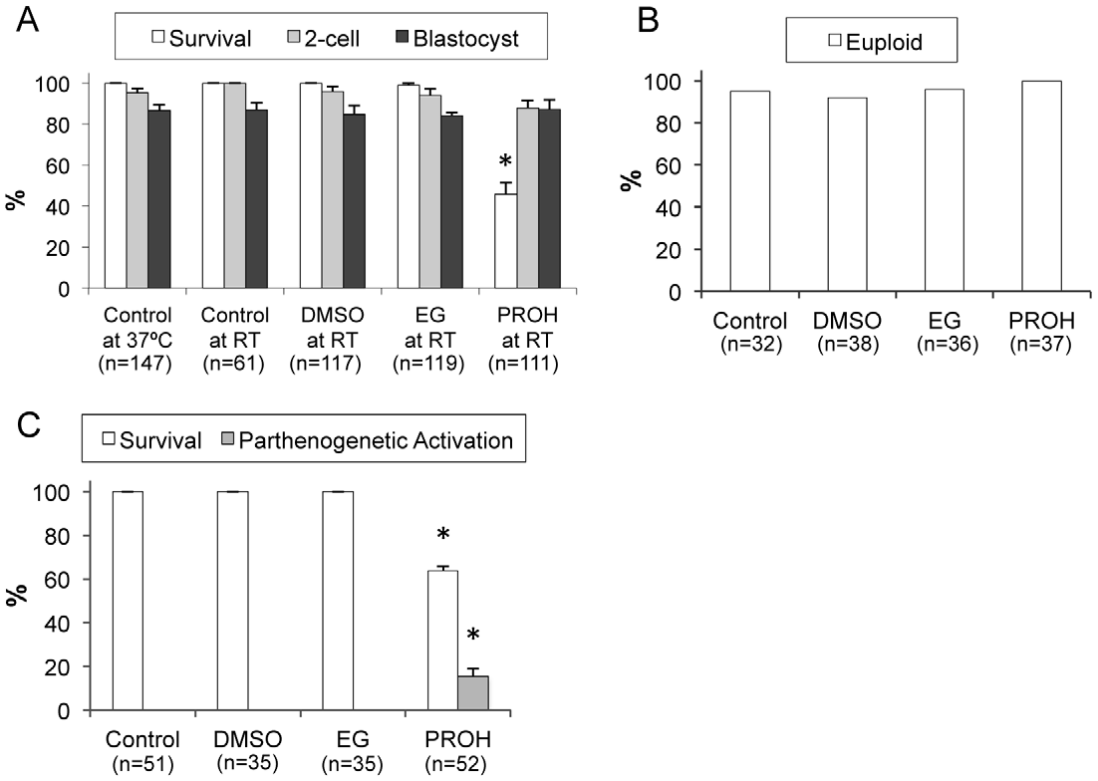
Toxicity of penetrating CPAs at room temperature. Ovulated mouse oocytes were exposed to a 1.5 M solution of each CPA at RT (∼23°C) for 15 minutes and evaluated for their (A) post-exposure survival, fertilization, and embryonic development, as well as for their (B) ploidy and (C) parthenogenetic activation. Data shown are mean±SEM except for the ploidy rates, which represent percentage of total number of euploid eggs. The total number of oocytes (n) used in each group was also shown. * denotes significant differences in survival and parthenogenetic activation (*p*<0.05).

This set of CPA exposure experiments was designed to compare the toxicity of penetrating CPAs from the perspective of slow cooling protocols. To this end, M II oocytes were exposed to a 1.5 M solution of each CPA for 15 minutes and subsequently analyzed along with controls. Some control oocytes were held at 23°C without exposure to any CPA to decouple the effect of cooling to RT. A total of 555 oocytes were used to elucidate the effect of the three CPAs on post-exposure survival, fertilization and development. The results are summarized in Fig. 1. Similar proportions of control oocytes kept at 37°C or exposed to RT were fertilized (95.4% and 100%, respectively) and developed to the blastocyst stage (86.5% and 86.9%, respectively), suggesting that keeping mouse oocytes at RT up to 30 min does not have a negative impact on fertilization and embryonic development. Similarly, exposure to 1.5 M DMSO or 1.5 M EG did not adversely affect morphological survival of the oocytes (100% and 99%, respectively) compared to controls at 37°C (100%) and RT (100%). In contrast, less than half (45.8%) of the oocytes survived the exposure to 1.5 M PROH at RT for 15 min, indicating the toxic effect of PROH. This survival rate after exposure to 1.5 M PROH was significantly lower than that for all the other groups. Among the surviving oocytes, the fertilization and embryonic development rates were similar in DMSO- (98.8% and 84.8%, respectively), EG- (94.1% and 84.0%, respectively), and PROH-exposure (88.0% and 87.3%, respectively) groups, and were comparable to controls.

To further probe the toxicity of the three penetrating CPAs, we also examined chromosomal abnormalities and parthenogenetic activation after CPA exposure using a total of 143 and 173 oocytes, respectively. Similar artificial activation rates were obtained in control (94%) and CPA-exposure (92–97%) groups. As shown in Fig. 1B, none of the three CPAs induced a significant increase in chromosomal abnormalities compared to controls. The euploidy rates remained high and were 92%, 96%, 100%, and 95% for DMSO, EG, PROH, and controls, respectively. However, the rate of parthenogenetic activation was significantly higher (15.3%) after exposure to 1.5 M PROH compared to that of the two other CPAs and controls (0% for all) as shown in Fig. 1C.

### Time- and Temperature-Dependence of CPA Toxicity

**Figure 2:**
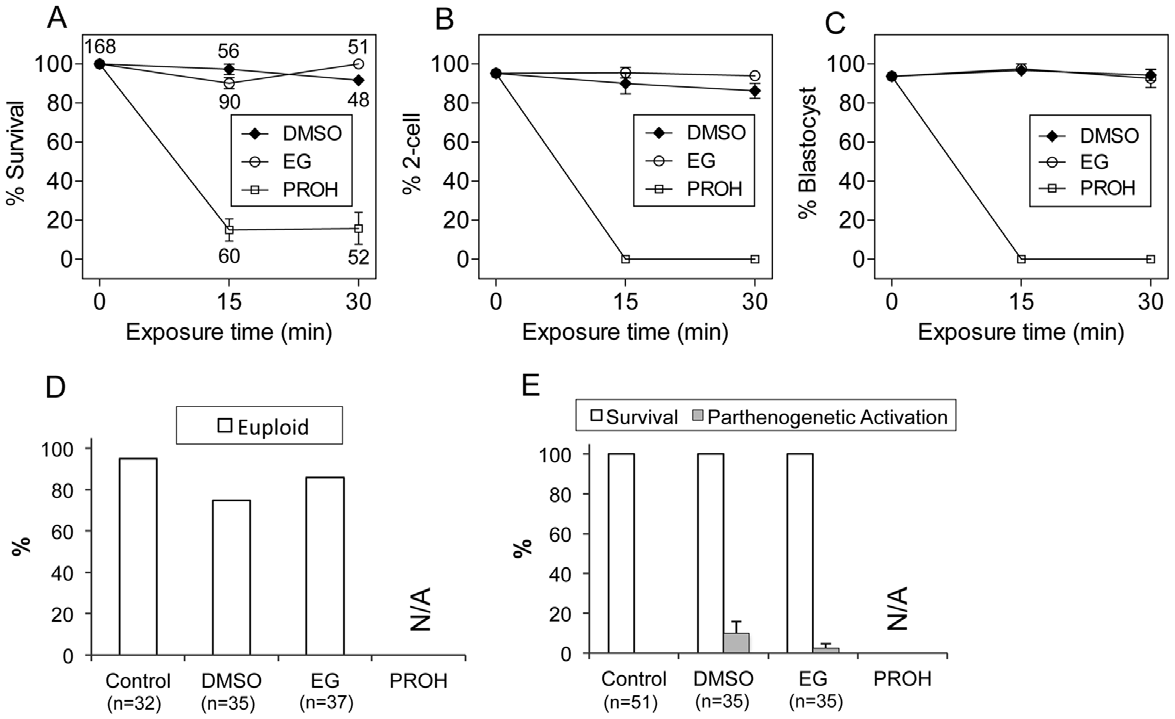
Time- and temperature-dependence of CPA toxicity. Ovulated mouse oocytes were exposed to a 1.5 M solution of each CPA at 37°C for 0, 15, and 30 minutes and evaluated for their (A) post-exposure survival, (B) fertilization, (C) embryonic development, (D) parthenogenetic activation, and (E) ploidy. Data shown are mean±SEM except for the ploidy rates, which represent percentage of total number of euploid eggs. The total number of oocytes (n) used in each group was also shown. The differences between the control, DMSO and EG groups were not significant while only a few oocytes survived exposure to 1.5 M PROH at 37°C. Therefore, parthenogenetic activation and ploidy were not evaluated in the PROH group. N/A: not applicable.

To elucidate time- and temperature-dependence of CPA toxicity, MII oocytes were first exposed to a 1.5 M solution of each CPA at 37°C for 0, 15, and 30 minutes, and then analyzed for their post-exposure survival, fertilization, and blastocyst rates, as well as for their chromosomal abnormalities, and parthenogenetic activation with respect to non-treated controls. A total of 750 oocytes were used for this set of experiments. The results are summarized in Fig. 2. Raising the exposure temperature from RT to 37°C further increased the toxic effect of PROH, leading to degeneration of the vast majority of oocytes (85.0% after 15 minutes and 84.2% after 30 minutes of exposure). Moreover, the surviving oocytes failed to fertilize, resulting in no embryonic development. In contrast, the toxicity of DMSO and EG at 37°C still remained insignificant after 15 minutes of exposure to their 1.5 M concentrations, with survival (97.3% and 90.2%, respectively), fertilization (90.0% and 95.6%, respectively), and blastocyst rates (96.7% and 97.6%, respectively) comparable to those of controls (100%, 95.3%, and 93.7%, respectively). Also, prolonging the exposure time to 30 minutes did not increase the toxic effect of DMSO and EG in terms of survival (91.7% and 100.0%, respectively), fertilization (86.3% and 94.0%, respectively), and embryonic development (94.3% and 92.7%, respectively). Similarly, exposure to DMSO and EG at 37°C for 30 minutes did not induce a significant increase in chromosomal abnormalities (25% and 14%, respectively) and parthenogenetic activation (10% and 2.3%, respectively) with respect to controls (5% and 0%, respectively).

### Dose-Dependence of CPA Toxicity

**Figure 3:**
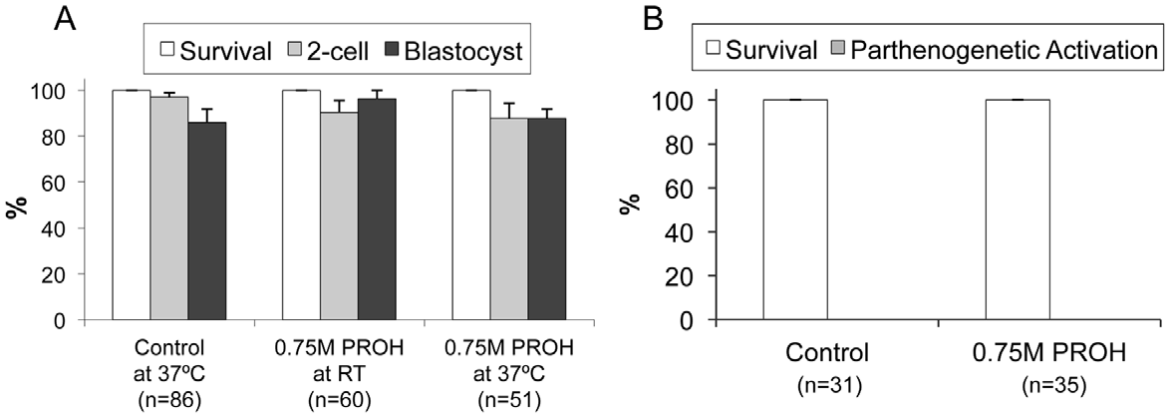
Dose-dependence of the toxicity of PROH. Ovulated mouse oocytes were exposed to a 0.75 M solution of PROH at both RT and 37°C for 15 minutes and then evaluated for their (A) post-exposure survival, fertilization, and embryonic development, as well as for their (B) parthenogenetic activation rate. Data shown are mean±SEM. The total number of oocytes (n) used in each group was also shown. There was no significant difference between the groups (*p*>0.05).

Based on the insignificant toxicity of DMSO and EG at their 1.5 M concentration (see the results above) and the substantial toxicity of higher concentrations (≥2 M) of all three CPAs even after a short exposure (3 min) at RT [28], it was not necessary to test high concentrations of none of the CPAs and lower concentrations of DMSO and EG. Therefore, we focused our experiments on PROH to see whether there was a dose-dependent decrease in its toxicity. To this end, we tested half (0.75 M) of its typical concentration at both RT and 37°C with respect to untreated controls using a total of 263 oocytes. As shown in Fig. 3, upon exposure to half of its typical slow cooling concentration, the toxicity of PROH completely disappeared, resulting in post-exposure survival (100%), fertilization (90,3%), and embryonic development (96.3%) rates similar to those of controls (100%, 97.2%, and 85.8%, respectively). Raising the exposure temperature to 37°C also did not induce any significant toxicity in terms of post-exposure survival (100%), fertilization (87.7%), and embryonic development (87.7%) compared to untreated controls. Furthermore, no parthenogenetic activation was observed after exposure to 0.75 M PROH at 37°C for 15 minutes, suggesting a strong dose-dependence of PROH toxicity. Chromosomal abnormalities were not tested because even higher concentrations of PROH (1.5 M) did not caused any toxicity.

### Avoidance of CPA Toxicity and Comparison of Post-Thaw Survival

**Figure 4:**
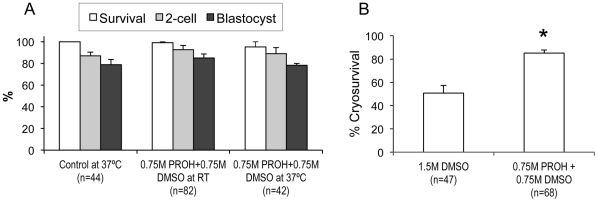
Avoidance of CPA toxicity and improvement of the cryosurvival. (A) Post-exposure survival, fertilization, and embryonic development. PROH and DMSO were combined using half (i.e., 0.75 M) of their typical concentrations to bring the total CPA concentration to a cryoprotective level without a significant toxic effect. Subsequently, ovulated mouse oocytes were exposed to a 1.5-M mixture of PROH and DMSO at both RT and 37°C for 15 minutes, and then were evaluated for their post-exposure survival, fertilization, and embryonic development rates. Data shown are mean±SEM. The total number of oocytes (n) used in each group was also shown. There was no significant difference between the groups (*p*>0.05). (B) Post-thaw survival rates. Ovulated mouse oocytes were loaded with either 0.75 M PROH+0.75 M DMSO or 1.5 M DMSO at RT for 15 minutes, and then subjected a freeze-thaw cycle to evaluate the cryoprotection of the combined 0.75-M concentrations of PROH and DMSO with respect to a commonly used concentration of DMSO alone. Data shown are mean±SEM. The total number of oocytes (n) used in each group was also shown. * denotes significant difference in the cryosurvival (*p*<0.001).

PROH is a preferred CPA [45], [46] as a result of its good glass-forming properties and high membrane permeation that results in less volumetric perturbations. Based on the encouraging results with 0.75 M PROH, we asked the question whether PROH can safely be used in slow cooling protocols by combining its lower concentration (0.75 M) with another penetrating CPA (e.g., 0.75 M DMSO) to bring the combined total CPA concentration to a cryoprotective level (i.e., 1.5 M) without increasing the overall CPA toxicity. To this end, mouse oocytes were exposed to a combined solution of 0.75 M PROH and 0.75 M DMSO at both RT and 37°C for 15 minutes, and their post-exposure survival, fertilization, and embryonic development rates were examined with respect to non-treated controls. A total of 168 oocytes were used for this set of experiments. As shown in Fig. 4A, exposure of M II oocytes to the combined 1.5 M concentration of PROH and DMSO at RT did not adversely affect their post-exposure survival (98.9%), fertilization (92.7%), and embryonic development (84.9%) compared to controls (100%, 87.0%, and 78.9%, respectively). When the exposure temperature was raised to 37°C, the survival (95.2%), fertilization (88.9%), and embryonic development (78.3%) rates were still comparable to controls, suggesting that a combination of two CPAs can be helpful to increase the total CPA concentration without increasing CPA toxicity.

Furthermore, we also tested the cryoprotective effect of the combined 0.75-M concentrations of PROH and DMSO with respect to a commonly used concentration (i.e., 1.5 M) of DMSO alone by subjecting a total of 115 M II oocytes to freezing and thawing in the presence of the respective CPA solutions. As shown in Fig. 4B, combining lower concentrations (i.e., 0.75 M) of PROH and DMSO not only avoids CPA toxicity but also significantly improves the cryosurvival rate (85.2%) in comparison to that (50.8%) of the widely used equivalent concentration (i.e., 1.5 M) of DMSO alone.

## Discussion

The toxicity of CPAs has been a limiting step for the use of high CPA concentrations, and thus for improvement of cryopreservation protocols. One strategy to deal with this issue is to employ less toxic but reasonably efficient CPAs. In the present study, we compared the toxicity of three commonly used penetrating CPAs (i.e., DMSO, EG, and PROH) to find a less toxic CPA for cryopreservation of mammalian oocytes. Our results involving survival, fertilization, embryonic development, as well as chromosomal abnormalities and parthenogenetic activation of mouse oocytes after exposure to each CPA at different temperatures for different durations show that DMSO and EG at their moderate concentrations (i.e., 1.5 M) are safer to use for oocyte cryopreservation than 1.5 M PROH. Nevertheless, the toxicity of PROH could also be avoided by combining its lower concentration (i.e., 0.75 M) with 0.75 M DMSO while keeping the total CPA concentration at a cryoprotective level. Moreover, this approach could also improve the cryosurvival, as demonstrated by our cryopreservation experiments in the present study.

Although there is no consensus on less or more toxic penetrating CPAs, published studies generally agree that the toxicity of penetrating CPAs increases with increased CPA concentration, higher exposure temperature, and longer exposure time. In the present study, extending the CPA exposure duration from 15 minutes to 30 minutes and raising the exposure temperature from 23°C to 37°C did not significantly increase the toxic effect of 1.5 M DMSO and 1.5 M EG on viability, fertilization, embryonic development, parthenogenetic activation, and chromosomal normality of mouse oocytes while the toxicity of 1.5 M PROH was significantly increased, as indicated by degeneration of the vast majority of PROH-exposed oocytes. These results suggest that the time- and temperature-dependence of CPA toxicity may become less pronounced depending on CPA type and concentration, although our findings do not exclude other toxic effects such as changes in the oocyte proteome [37]. Overall, our results are assuring from the perspective of slow cooling protocols, which typically use CPA concentrations around 1.5 M. However, higher concentrations of these CPAs induce a significant adverse effect even within 3 minutes of exposure at RT [28]. Therefore, vitrification protocols involving high concentrations of CPAs require a great deal of attention to the timing of CPA exposure and handling of oocytes.

Earlier studies showed that cooling and exposure to CPAs can independently induce depolymerization/disruption of the oocyte cytoskeleton such as microfilaments and meiotic spindle microtubules, potentially leading to dispersion and improper segregation of chromosomes, failure in polar body formation, and thus chromosomal abnormalities [47], [48], [49]. In fact, increased chromosomal abnormalities have been reported after oocyte cryopreservation [12], [50], [51]. In the present study, no significant increase in chromosomal abnormalities after CPA exposure suggests that mouse oocytes were able to recover from CPA-induced damage to the oocyte cytoskeleton and spindle microtubules. This is encouraging from the safety perspective although combining the CPA exposure with cooling/freezing stresses may complicate things. Also, oocytes from some species seem to have less competence to restore a damaged spindle [48], [52]. Therefore, additional measures might be useful to protect the meiotic spindle in such oocytes with limited recovery competence.

Parthenogenetic activation of ovulated oocytes may occur as result of a number of chemical and physical stimuli, such as exposure to ethanol [53], Ca^2+^ ionophore [54], Sr^2+^ [55], hyaluroidase [55], electric pulse [55], and cooling [55]. It has also been shown that exposure of mouse M II oocytes to 1.5 M PROH significantly increases the parthenogenetic activation rate [10], [11]. The results of the present study are consistent with previous findings. In contrast, no significant increase in the parthenogenetic activation rate was observed after exposure of rabbit oocytes to 1.5 M PROH at RT for 30 minutes, suggesting interspecies differences [49]. Human oocytes also seem to be somewhat resistant to parthenogenetic activation. Exposure to 1.5 M PROH alone was not sufficient to significantly induce parthenogenetic activation while a combination of PROH exposure with a freeze-thaw cycle significantly increased the number of parthenogenetically activated human oocytes [56]. Together these findings suggest that the extent of CPA toxicity may vary depending on species. It has been suggested that the ability of certain compounds such as ethanol and PROH to artificially activate M II oocytes might be due to the presence of –OH groups in their structure [10], [11]. However, EG, which is chemically closely related to PROH and also contains two –OH groups, did not cause parthenogenetic activation in the present study, suggesting that the presence of –OH groups alone is not sufficient to induce a significant increase in parthenogenetic activation.

Despite several published studies on CPA toxicity, its mechanism of action still remains poorly understood. Hydrophobic interactions between CPAs and proteins [57], and the extent of hydrogen binding between CPAs and water molecules [27] have been proposed to explain CPA toxicity. It has also been shown that CPAs change the intracellular pH [58], cause intracellular Ca^2+^ release [59], and induce formaldehyde formation in cryopreservation medium [60]. Further, it has been questioned whether CPA toxicity is related to osmotic stresses that occur during addition and removal of CPAs. Although such osmotic stresses can lead to cytotoxicity [28], experimental evidence suggests that CPAs clearly induce chemical toxicity [28], [61]. Among the tested CPAs, PROH permeates into mouse oocytes faster than DMSO and EG, and thus causes less osmotic stresses [62]. Yet, only PROH showed a significant toxic effect in the present study. Therefore, it is unlikely that the toxicity of PROH observed here is due to osmotic stresses. This notion is also supported by an earlier study showing that stepwise addition and removal of 1.5 M PROH does not reduce its toxic effect in terms of oocyte degeneration and parthenogenetic activation [10].

In the present study, we assessed the fertilization rate of control and CPA-exposed oocytes based on their cleavage to the two-cell stage. On the other hand, our experiments on parthenogenetic activation showed that exposure of M II oocytes to 1.5 M PROH at RT and 1.5-M concentrations of DMSO and EG at 37°C can induce parthenogenetic activation up to 15%, 10%, and 3%, respectively. Hence, it is possible that a small proportion of the two-cell embryos might have been parthenogenetically activated, which should be taken into account when interpreting these particular fertilization results. It is also important to note that in terms of blastocyst quality, we have not observed any significant morphological difference between the control and CPA exposure groups. However, long-term effects of CPAs beyond the blastocyst stage require further comprehensive studies.

In conclusion, DMSO and EG are safer than PROH in terms of minimization of CPA toxicity in slow cooling protocols. However, PROH can also be used without significant toxicity by combining its lower concentration (0.75 M) with another penetrating CPA to bring the total CPA concentration to a cryoprotective level (i.e., 1.5 M). Considering the different protective actions of each CPA [63], [64], this approach may also be helpful to improve the overall cryoprotection, as shown in the present study.
